# Iodine-131 induces ferroptosis and synergizes with sulfasalazine in differentiated thyroid cancer cells via suppressing *SLC7A11*

**DOI:** 10.3389/fonc.2025.1580828

**Published:** 2025-05-19

**Authors:** Li Ling, Jinhe Zhang, Xiao Zhang, Peiqi Wang, Mingjun Ma, Bingling Yin

**Affiliations:** ^1^ Graduate School, Guangzhou University of Chinese Medicine, Guangzhou, Guangdong, China; ^2^ Department of Nuclear Medicine, General Hospital of Southern Theater Command, Guangzhou, Guangdong, China

**Keywords:** differentiated thyroid cancer, ferroptosis, Iodine-131, sulfasalazine, SLC7A11 (xCT)

## Abstract

Iodine-131 (^131^I) plays a key role in the treatment of differentiated thyroid cancer (DTC). Ferroptosis represents a form of regulated cell death that is distinct from necrosis and apoptosis, constituting a unique mode of programmed cell death. In this study, we aimed to ascertain the potential of ^131^I to trigger ferroptosis in DTC and to assess the synergistic therapeutic impact of combining ^131^I with sulfasalazine (SAS), a ferroptosis inducer, in the context of DTC. The FTC-133 and TPC-1 cell lines were employed to evaluate the impact of ^131^I and SAS on cellular functions. Ferrostatin-1 (Fer-1) reversed the cell viability and colony formation ability inhibited by ^131^I. ^131^I led to an elevation in the levels of malondialdehyde (MDA), reactive oxygen species (ROS), and lipid peroxidation. DTC cells exposed to ^131^I displayed characteristic ferroptotic ultrastructure, featuring shrunken mitochondria with increased membrane density. Concurrently, there was a reduction in the content of glutathione (GSH), as well as a downregulation of the expression levels of glutathione peroxidase 4 (*GPX4*) and solute carrier family 7 member 11 (*SLC7A11*) in the cells treated with ^131^I. The CI values for the combination of SAS and ^131^I in DTC cells were less than 1, demonstrating that SAS synergized with ^131^I. Moreover, the combination of SAS and ^131^I significantly increased the MDA levels and lipid peroxidation, decreased the GSH levels, and suppressed the expression of SLC7A11 and GPX4, while *SLC7A11* knockdown significantly enhanced ferroptosis-related markers in DTC cells. Animal experiments demonstrated that SAS synergized with ^131^I resulted in notable decreases in tumor volume and weight. Furthermore, immunohistochemical analyses revealed that the combination of ^131^I and SAS significantly downregulated the expression of GPX4 and SLC7A11 *in vivo*. Taken together, our results suggest that ^131^I may induce lipid peroxidation and ferroptosis, and demonstrate the potential for a synergistic therapeutic effect when ^131^I is combined with SAS in the treatment of DTC.

## Introduction

Thyroid cancer is the most prevalent type of endocrine cancer globally. In 2022, it ranked as the seventh most frequently diagnosed cancer worldwide, with its incidence steadily increasing annually (1, 2). The predominant subtype, differentiated thyroid cancer (DTC), generally demonstrates favorable prognosis following standard treatments including surgery and radioactive iodine (RAI) therapy (3). RAI is primarily utilized for three clinical purposes: remnant ablation, adjuvant therapy, and therapeutic intervention in metastatic disease (4, 5).

Ferroptosis, a regulated form of cell death distinct from necrosis and apoptosis in morphology, biochemistry, and genetics, was first conceptualized by Dixon et al. in 2012 (6). This iron-dependent process is driven by lipid peroxidation and has been implicated in both physiological processes and pathological conditions ranging from ischemia-reperfusion injury to neurodegenerative diseases (7–10). Although ferroptosis functions as a natural tumor-suppressive mechanism, its activity is often suppressed in cancer cells (11–13). Given this tumor-suppressive paradox, reactivating ferroptosis represents a promising therapeutic strategy for diverse malignancies. Studies have confirmed that conventional cancer therapies, including radiotherapy, can induce ferroptosis (14, 15). However, the ability of ^131^I—a widely used internal radiotherapy agent in DTC—to trigger ferroptosis remains underexplored.

*GPX4*, a central inhibitor of ferroptosis, suppresses lipid peroxidation by catalyzing the reduction of lipid hydroperoxides to lipid alcohols using glutathione (GSH) as a cofactor (16). *SLC7A11* (*xCT*) is a cystine/glutamate antiporter that plays a crucial role in transporting cystine into cells, which is essential for the production of cysteine—a rate-limiting precursor for GSH synthesis (17). Consequently, pharmacological inhibition of *SLC7A11* promotes ferroptosis through dual mechanisms: depleting intracellular GSH and indirectly impairing *GPX4*-mediated lipid repair (18). Recent studies demonstrated that ionizing radiation triggers ferroptosis by downregulating SLC7A11 expression in solid tumors (15). Sulfasalazine (SAS), an FDA-approved drug for inflammatory bowel disease, has recently been repurposed as a ferroptosis inducer that directly inhibits SLC7A11 activity, thereby accelerating lipid peroxidation (19). Notably, SAS exhibits radiosensitizing effects by synergistically reducing GSH levels to amplify radiation-induced ferroptosis (20, 21). Moreover, *SLC7A11* overexpression in thyroid cancer tissues suggests its inhibition could offer therapeutic benefits (22–25). Integrating the dual roles of *SLC7A11* in ferroptosis regulation and thyroid cancer progression, we hypothesize that combining SAS with ^131^I may synergistically enhance treatment efficacy through concerted *SLC7A11* inhibition.

This study aims to investigate the involvement of ferroptosis in ^131^I-induced cell death in DTC and evaluate the therapeutic potential of combining SAS with ^131^I.

## Materials and methods

### Cell culture and reagents

The normal thyroid cell lines Nthy-ori3-1, kindly provided by the Department of Nuclear Medicine, First Affiliated Hospital of Guangdong Pharmaceutical University, and the human thyroid cancer cell lines TPC-1 and FTC-133, obtained from Zhongqiao Xinzhou Biological Co., Ltd. (Shanghai, China), were cultured in RPMI-1640 medium supplemented with 10% fetal bovine serum (BI, Shanghai, China) and 1% penicillin/streptomycin (BI, Shanghai, China). Cells were maintained at 37°C in a humidified 5% CO_2_ atmosphere. Ferrostatin-1 (HY-100579) and sulfasalazine (HY-14655) were procured from MedChemExpress (Monmouth Junction, NJ, USA). Z-VAD-FMK (T7020) and necrostatin-1 (T1847) were obtained from TargetMol (Boston, MA, USA). Sodium iodide-131 (Na¹³¹I) was purchased from HTA Co., Ltd. (Beijing, China).

### CCK-8 assay

Cell viability of FTC-133 and TPC-1 cells treated with ^131^I, Ferrostatin-1 (Fer-1), Z-VAD-FMK, necrostatin-1, or SAS was quantified using a CCK-8 assay kit (GIPBIO, Montclair, NJ, USA). Cells were seeded into 96-well plates at a density of 5,000 cells/well and treated with compounds according to experimental protocols. After treatment, the medium in each well was replaced with 100 μL of fresh RPMI-1640 containing 10 μL CCK-8 reagent. Following a 3-hour incubation at 37°C under light-protected conditions, absorbance was measured at 450 nm using a microplate reader (Thermo Fisher Scientific, Waltham, MA, USA). Cell viability (%) was calculated using the formula:


$$\text{Viability }\left(\%\right)=\left[\left({\text{OD}}_{\text{treated}}–{\text{OD}}_{\text{blank}}\right)/\left({\text{OD}}_{\text{untreated}}–{\text{OD}}_{\text{blank}}\right)\right]\;\times \;100\%$$

$${\text{OD}}_{\text{blank}}\text{ refers to wells containing medium without cells}$$

### Colony-formation assay

Cells were seeded in 6-well plates at a density of 700 cells/well. The combination group and Fer-1 monotherapy group were pre-treated with 1 μM Fer-1 for 4 hours; subsequently, the combination group and ^131^I monotherapy group were exposed to ^131^I (50 μCi/mL). Cells were cultured in a humidified 5% CO_2_ incubator at 37°C until visible colonies formed. Colonies were fixed with 4% paraformaldehyde (PFA) and stained with 0.5% crystal violet (Biosharp, Hefei, Anhui, China) for 30 minutes at room temperature. Colonies in each well were imaged and quantified using ImageJ software (National Institutes of Health, Bethesda, MD, USA).

### Transmission electron microscopy

Cells cultured in 100-mm dishes were exposed to 131I (50 μCi/mL) for 48 h. After treatment, cells were fixed with 2.5% glutaraldehyde and 2% paraformaldehyde in 0.1 M phosphate buffer (pH 7.4) at 4°C overnight. Subsequently, cells were washed three times with phosphate buffer and post-fixed in 2% osmium tetroxide at room temperature for 1 hour. Following dehydration through a graded acetone series (50%, 70%, 90%, 100%), cells were stained with 1% uranyl acetate, embedded in Epon 812 resin (SPI Supplies, West Chester, PA, USA), and polymerized at 60°C for 48 hours. Ultrathin sections (70 nm) were prepared using an ultramicrotome (Leica EM UC7, Germany) and examined with a transmission electron microscope (HT7800, Hitachi, Tokyo, Japan).

### GSH measurement

Intracellular GSH levels were determined using a GSH assay kit (Solarbio Science & Technology Co., Ltd., Beijing, China) according to the manufacturer’s protocol. Briefly, cells were seeded in 6-well at a density of 1×10^5^ cells/well and treated according to experimental protocols. After treatment, cells were harvested and divided into two equal aliquots: one for protein quantification via the bicinchoninic acid (BCA) assay and the other for GSH analysis. For GSH detection, cells were lysed by sonication after adding Reagent I from the kit. The lysate was centrifuged at 12,000 × g for 10 minutes at 4°C to collect the supernatant. Reagents II and III were sequentially added to the supernatant, mixed thoroughly, and incubated at 25°C for 5 minutes. Absorbance at 412 nm was measured using a microplate reader (Thermo Fisher Scientific, Waltham, MA, USA).

### Malondialdehyde measurement

Intracellular MDA levels were quantified using an MDA assay kit (Beyotime Biotechnology, Shanghai, China) based on the thiobarbituric acid (TBA) reaction. Cells were seeded in 6-well plates at a density of 1×10^5^ cells/well and treated according to experimental protocols. After treatment, cells were harvested, lysed, and centrifuged to collect the supernatant. After protein quantification of the lysates, the MDA working solution was added, and the mixture was heated at 100°C for 15 minutes. The samples were then centrifuged at 1,000 × g for 10 minutes at 4°C. Absorbance of the supernatant was measured at 532 nm using a microplate reader (Thermo Fisher Scientific, Waltham, MA, USA).

### ROS detection

Intracellular reactive oxygen species (ROS) levels were detected using the fluorescent probe 2’,7’-dichlorodihydrofluorescein diacetate (DCFH-DA). Cells were seeded in 6-well plates at a density of 1 × 10^5^ cells/well. After cell adhesion, the combination group and Fer-1 monotherapy group were pre-treated with 1 μM Fer-1 for 4 hours; subsequently, the combination group and ^131^I monotherapy group were exposed to ^131^I (50 μCi/mL) for 48 h. After treatment, cells were incubated with 10 μM DCFH-DA (prepared in serum-free RPMI-1640) under light-protected conditions for 30 minutes. After washing three times with phosphate-buffered saline (PBS), fluorescence images were acquired using a fluorescence microscope (Axio Observer 7, Carl Zeiss AG, Oberkochen, Germany).

### Lipid peroxidation measurement

Cells (2×10^3^ cells/well) were seeded in 24-well plates and treated according to experimental groups. Lipid peroxidation levels were quantified using the C11-BODIPY 581/591 fluorescent probe (Beyotime Biotechnology Co., Ltd., Shanghai, China) according to the manufacturer’s protocol. Briefly, cells were incubated with 5 μM C11-BODIPY 581/591 under light-protected conditions for 30 minutes. Fluorescence intensity was measured using a microplate reader (Fluoroskan Microplate Reader, Thermo Fisher Scientific, Waltham, MA, USA) (reduced form: Ex/Em 581/591 nm; oxidized form: Ex/Em 488/510 nm). The results were expressed as the ratio of oxidized to reduced fluorescence intensity.

### Reverse transcription-quantitative polymerase chain reaction

Total RNA was isolated from FTC-133 and TPC-1 cells using TRIzol™ reagent (Thermo Fisher Scientific, Waltham, MA, USA). Complementary DNA (cDNA) was synthesized from 1 μg RNA using SuperScript™ II Reverse Transcriptase (Vazyme Biotech Co., Ltd., Nanjing, Jiangsu, China). RT-qPCR amplification was conducted on a LineGene Pro Real-Time PCR System (Bioer Technology, Hangzhou, China) with the following cycling conditions: 95°C for 5 min, followed by 40 cycles of 95°C for 10 s and 60°C for 30 s. mRNA expression levels were normalized to the endogenous control gene *GAPDH*. Primer sequences were as follows:

Homo-GPX4-F: 5’-ACATGGTTAACCTGGACAAGT     ACCG-3’

Homo-GPX4-R: 5’-GGTCGACGAGCTGAGTGTAG     TTTAC-3’

Homo-SLC7A11-F: 5’-TGGAAGTCTTTGGTCCATTACC     AGC-3’

Homo-SLC7A11-R: 5’-GGTTCCAGAATGTAGCGTCCA     AATG-3’

Homo-GAPDH-F: 5’-ACAGCCTCAAGATCATCAGCA-3’

Homo-GAPDH-R: 5’-ATGAGTCCTTCCACGATACCA-3’.

### Western blotting

Proteins were extracted from FTC-133 and TPC-1 cells of each experimental group using RIPA lysis buffer (Biosharp Life Sciences, Anhui, China). Protein concentrations were determined using a BCA Protein Assay Kit (Thermo Fisher Scientific, Waltham, MA, USA). Equal amounts of protein (40 μg per lane) were separated by SDS-PAGE and transferred onto polyvinylidene difluoride (PVDF) membranes (Merck Millipore, Burlington, MA, USA). Membranes were blocked with 5% non-fat milk for 1 h at room temperature, followed by overnight incubation at 4°C with primary antibodies. After washing, membranes were incubated with HRP-conjugated secondary antibodies for 1 h. Protein bands were visualized using an Enhanced Chemiluminescent (ECL) Substrate Kit (Abbkine Scientific Co., Ltd., Wuhan, Hubei, China) and imaged on a ChemiDoc MP Imaging System (Bio-Rad Laboratories, Hercules, CA, USA). Antibody details: Anti-SLC7A11 (1:1,000; HUABIO, Hangzhou, Zhejiang, China), Anti-GPX4 (1:1,000; Cell Signaling Technology, Danvers, MA, USA), Anti-GAPDH (1:10,000; HUABIO, Hangzhou, Zhejiang, China), HRP-conjugated Goat Anti-Rabbit IgG (1:10,000; HUABIO, Hangzhou, Zhejiang, China).

### siRNA transfection

*SLC7A11*-targeting siRNAs were synthesized by IGE Biotechnology Co., Ltd. (Guangzhou, Guangdong, China). Transient transfection was performed using siRNA-Mate Transfection Reagent (GenePharma Co., Ltd., Shanghai, China) following the manufacturer’s instructions. FTC-133 cells were transfected with siRNA-*SLC7A11*-1(5’-GAAGAUAAGUUAUGAACUA-3’). TPC-1 cells were transfected with siRNA-*SLC7A11*-2 (5’-GGAAGAGAUUCAAGUAUUA-3’). Cells were harvested 48 h post-transfection for downstream analyses.

### Animal experiment

All animal experiments were conducted in accordance with the National Guidelines for Experimental Animal Welfare (the Ministry of Science and Technology of People’s Republic Laboratory Animals 2006-09-30) at the Animal Experiment Center of General Hospital of Southern Theater Command (Guangzhou, China). The animal experiments were approved by the Institutional Animal Care and Use Committee of General Hospital of Southern Theater Command. Sixteen BALB/C nude mice (3-4 weeks, 16-18 g) purchased from Guangzhou Ruige Biological Technology Co., Ltd (Guangzhou, China) were used for the TPC-1 cell xenografts. One week after adaptive feeding, the mice were randomly divided into four groups: control group (nude mice were administered an equivalent volume of saline as the other group), ^131^I group (nude mice were administered 7.4 MBq of ^131^I once every three days), SAS group (nude mice received 8 mg/kg SAS daily) and combination group (nude mice were administered 7.4 MBq of ^131^I once every three days and received 8 mg/kg SAS daily). After three courses of ^131^I treatment, mice were euthanized by cervical dislocation. Tumor size was calculated in accordance with the following formula: tumor volume = 1/2 × A × B^2^, where A and B represent the longest and shortest tumor diameters, respectively.

### Immunohistochemistry

Tumor tissues removed from mice models were fixed in 4% buffered formalin and embedded in paraffin. After the tumor tissue was sectioned into 5 μm thickness, hematoxylin-eosin staining was performed on the sections. For the purpose of immunohistochemical staining, the primary antibodies, including GPX4 (1:500, CST, USA) and SLC7A11 (1:500, HUABIO, Hangzhou, China), were incubated at 4°C overnight. Images were scanned using 3DHISTECH (PANNORAMIC MIDI, Budapest, Hungary).

### Statistical analysis

Quantitative data are presented as mean ± standard deviation (SD) derived from at least three independent biological replicates. Intergroup differences were analyzed by Student’s unpaired t-test (two groups) or one-way analysis of variance (ANOVA) with Tukey’s *post hoc* test (three or more groups). All analyses were performed using SPSS v26.0 (IBM Corp., Armonk, NY, USA). Statistical significance was defined as follows: p < 0.05 (*), p < 0.01 (**), and p < 0.001 (***); nonsignificant (ns) indicates p ≥ 0.05.

## Results

### Fer-1 alleviates cell viability and colony formation ability suppressed by ^131^I in DTC cells

**Figure 1:**
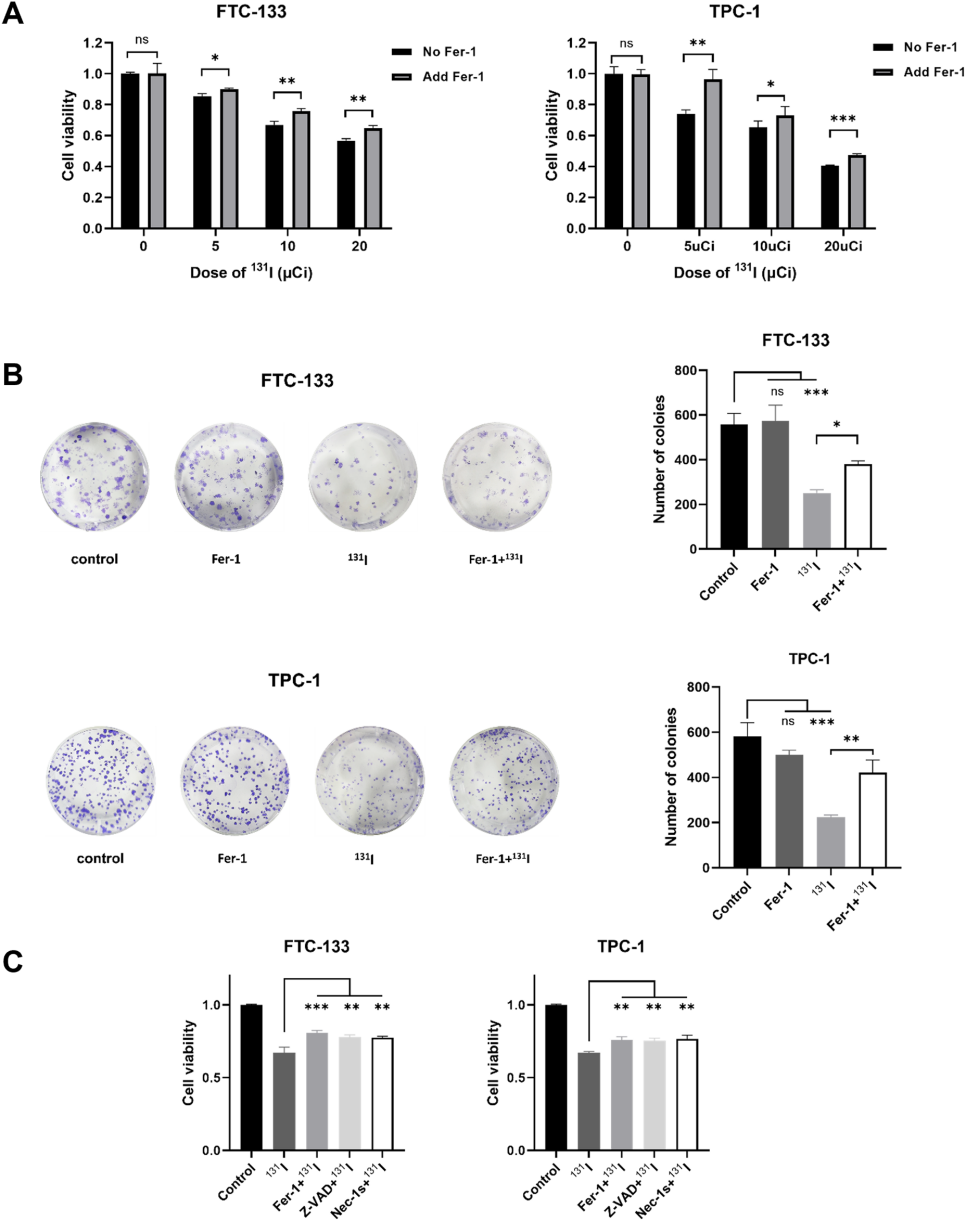
Fer-1 alleviates cell viability and colony formation ability suppressed by ^131^I in DTC cells. **(A)** Cell viability of FTC-133 and TPC-1 cells that were pretreated with 1 μM Fer-1 for 4 h followed by treatment of 5, 10, 20 μCi ^131^I for 48 h. **(B)** Colony formation ability of FTC-133 and TPC-1 cells that were pretreated with 1 μM Fer-1 for 4 h followed by treatment of 50 μCi ^131^I. **(C)** Cell viability of FTC-133 and TPC-1 cells that were pretreated with 1 μM Fer-1, 5 μM Necrostatin-1s or 10 μM Z-VAD-fmk for 4 h followed by treatment of 10 μCi ^131^I for 48 h. Images are representative of at least three independent experiments.

Ionizing radiation has been shown to trigger ferroptosis in cancer cells (14). Given that ^131^I emits both β- and γ-radiation, we hypothesized it could induce ferroptosis in DTC cells. To test this hypothesis, we first examined the effects of the ferroptosis inhibitor Fer-1 on FTC-133 and TPC-1 cells exposed to grade doses of ^131^I. Fer-1 significantly attenuated ^131^I-induced cytotoxicity, manifested by increased cell viability (**Figure 1A**). Colony formation assays corroborated these findings, demonstrating that Fer-1 alleviated ^131^I-mediated suppression of clonogenic capacity in both cell lines (**Figure 1B**). To elucidate the contribution of ferroptosis to ^131^I-induced cell death, we performed comparative analyses of Fer-1 with apoptosis inhibitor ZVAD-FMK and necroptosis inhibitor necrostatin-1 (Nec-1s). All three inhibitors partially restored viability in ^131^I-treated DTC cells (**Figure 1C**), indicating co-activation of ferroptotic, apoptotic, and necroptotic pathway. Based on these findings, we focused subsequent investigations on ferroptosis mechanisms in ^131^I-treated DTC cells.

### Biochemical hallmarks of ferroptosis are observed in ^131^I-treated DTC cells

**Figure 2:**
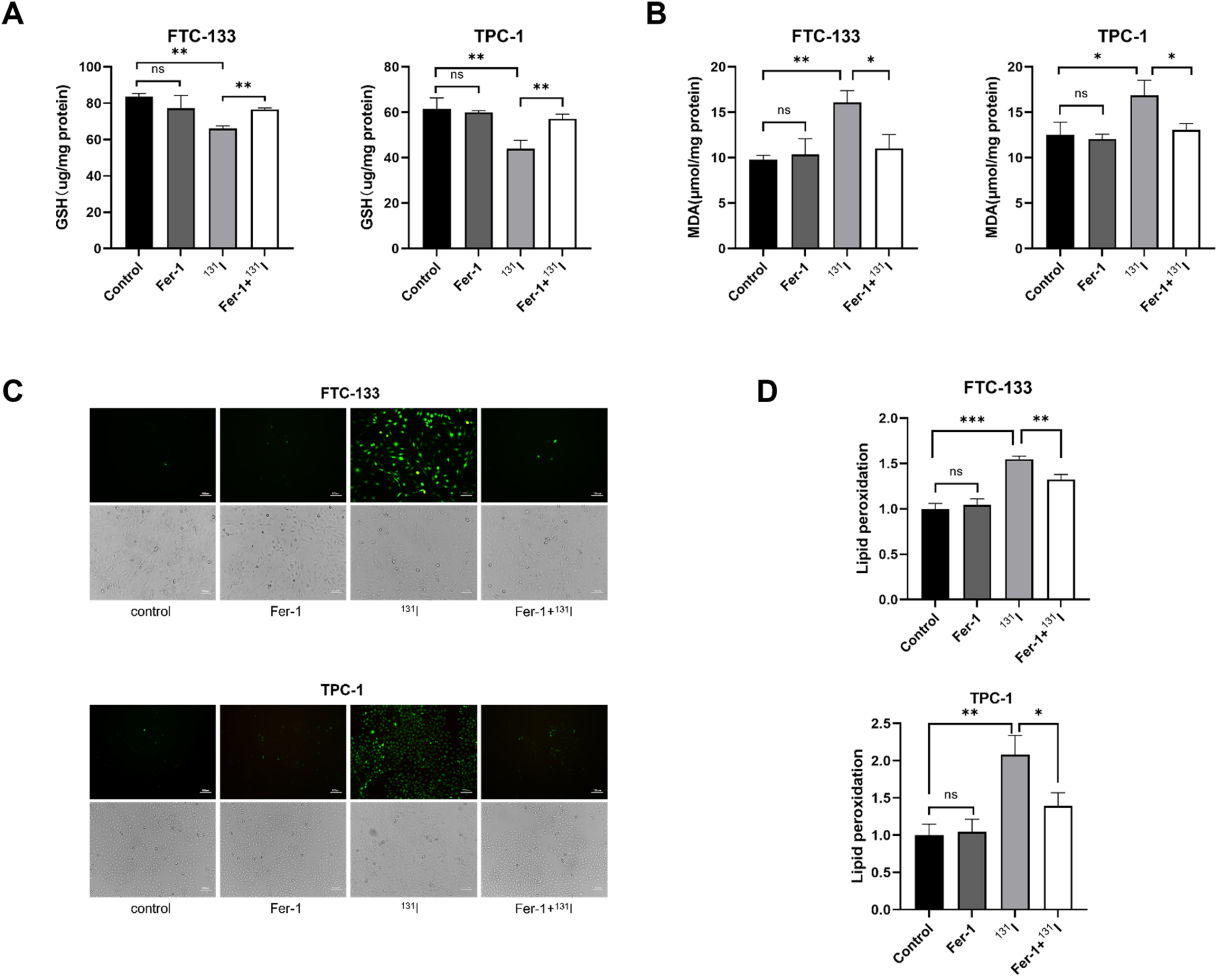
Ferroptosis morphological and genetic signatures are observed in ^131^I-treated DTC cells. **(A)** The GSH levels of FTC-133 and TPC-1 cells pretreated with 1 μM Fer-1 for 4 h followed by treatment of 50 μCi ^131^I for 48 h. **(B)** The MDA levels of FTC-133 and TPC-1 cells pretreated with 1 μM Fer-1 for 4 h followed by treatment of 50 μCi ^131^I for 48 h. **(C)** The ROS levels of FTC-133 and TPC-1 cells pretreated with 1 μM Fer-1 for 4 h followed by treatment of 50 μCi ^131^I for 48 h. Scale bar = 100 μm. **(D)** The lipid peroxidation levels of FTC-133 and TPC-1 cells pretreated with 1 μM Fer-1 for 4 h followed by treatment of 50 μCi ^131^I for 48 h. Images are representative of at least three independent experiments.

We systematically evaluated ferroptosis-associated biochemical alterations in DTC cells following ^131^I exposure. GSH levels were first quantified in FTC-133 and TPC-1 cells, revealing significant depletion upon ^131^I treatment (**Figure 2A**). Consistent with ferroptosis characteristics, MDA-a lipid peroxidation byproduct indicative of ferroptosis (26) - showed marked elevation in ^131^I-treated cells versus controls (**Figure 2B**). ROS production and lipid peroxidation were also substantially amplified in both cell lines post-^131^I exposure (**Figures 2C, D**). Notably, co-treatment with Fer-1 effectively abrogated ^131^I-induced perturbations in GSH, MDA, ROS, and lipid peroxidation markers (**Figures 2A-D**). These data collectively confirm ^131^I-triggered ferroptosis activation in DTC cells through canonical biochemical pathways.

### Ferroptosis morphological and genetic signatures are observed in ^131^I-treated DTC cells

**Figure 3:**
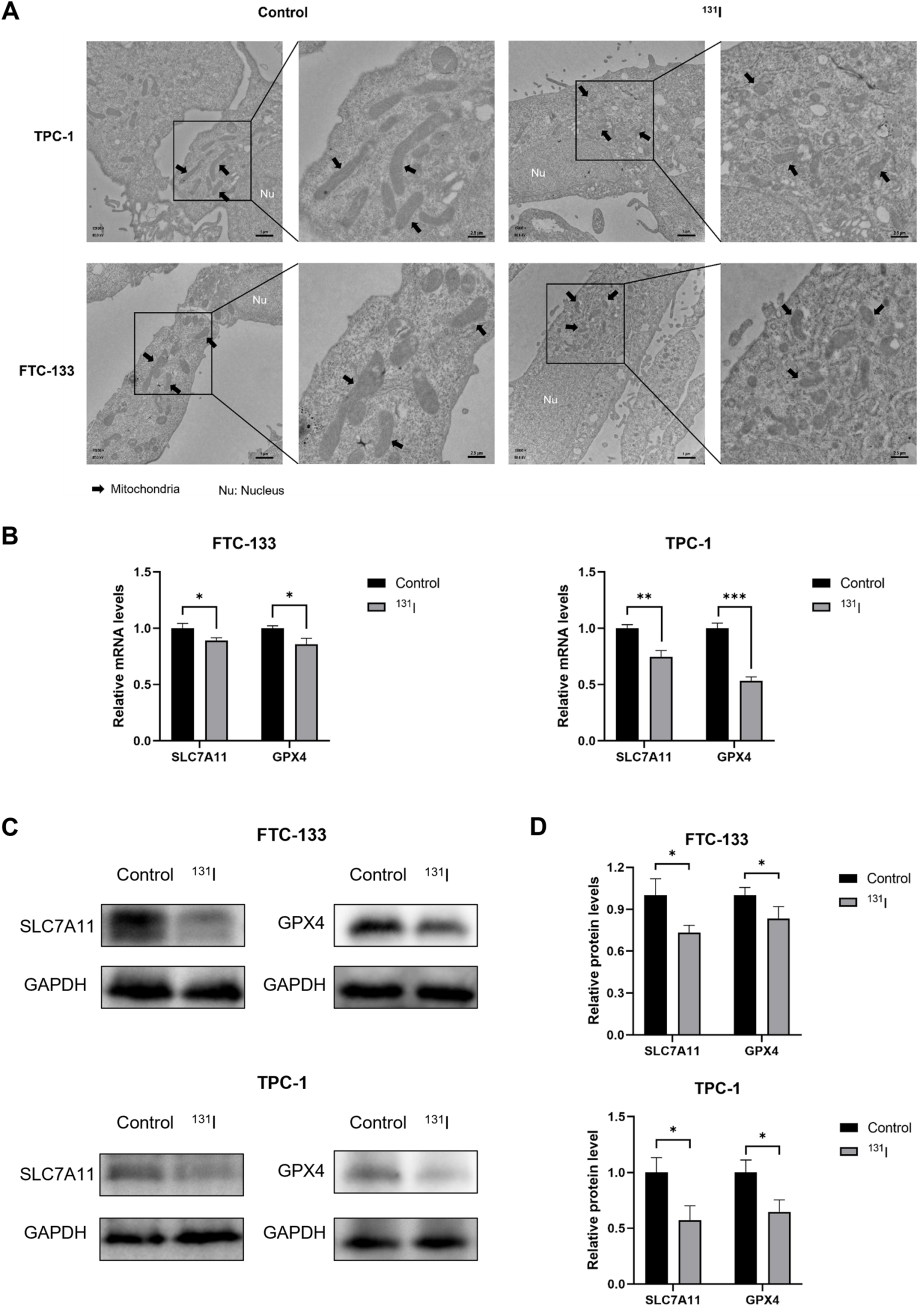
Morphological markers and Genetic hallmarks of ferroptosis were observed in ^131^I -treated DTC cells. **(A)** Transmission electron microscopy images of FTC-133 and TPC-1 cells with or without ^131^I treatment. Nu, nucleus. Scale bars: left, 1 μm, right, 2.5 μm. **(B)** The relative *SLC7A11* and *GPX4* mRNA expression levels of FTC-133 and TPC-1 cells with or without ^131^I treatment. **(C)** The relative *SLC7A11* and *GPX4* protein expression levels of FTC-133 and TPC-1 cells with or without ^131^I. **(D)** The quantitative graph of relative protein expression of *SLC7A11* and *GPX4* using Image J. Images are representative of at least three independent experiments.

Ferroptosis is characterized by distinct ultrastructural features that distinguish it from apoptosis and necroptosis. To systematically characterize these morphological changes, we performed TEM on ^131^I-treated DTC cells. Notably, FTC-133 and TPC-1 cells exposed to ^131^I displayed pathognomonic ferroptotic ultrastructure, featuring shrunken mitochondria with increased membrane density (27) (**Figure 3A**).

To e elucidate ferroptosis-related molecular mechanisms, we quantified *GPX4* and *SLC7A11* expression at both transcriptional and translational levels. RT-qPCR analyses revealed that ^131^I treatment significantly downregulated *GPX4* and *SLC7A11* mRNA levels relative to untreated controls (**Figure 3B**). Protein expression paralleled transcriptional changes, with marked reductions in GPX4 and SLC7A11 observed by western blot (**Figures 3C, D**). These results collectively demonstrate that ^131^I triggers ferroptosis in DTC cells through coordinated suppression of *GPX4* and *SLC7A11* and induces mitochondrial remodeling characteristic of ferroptosis in DTC cells.

### SAS synergizes with ^131^I to promote DTC cells death

**Figure 4:**
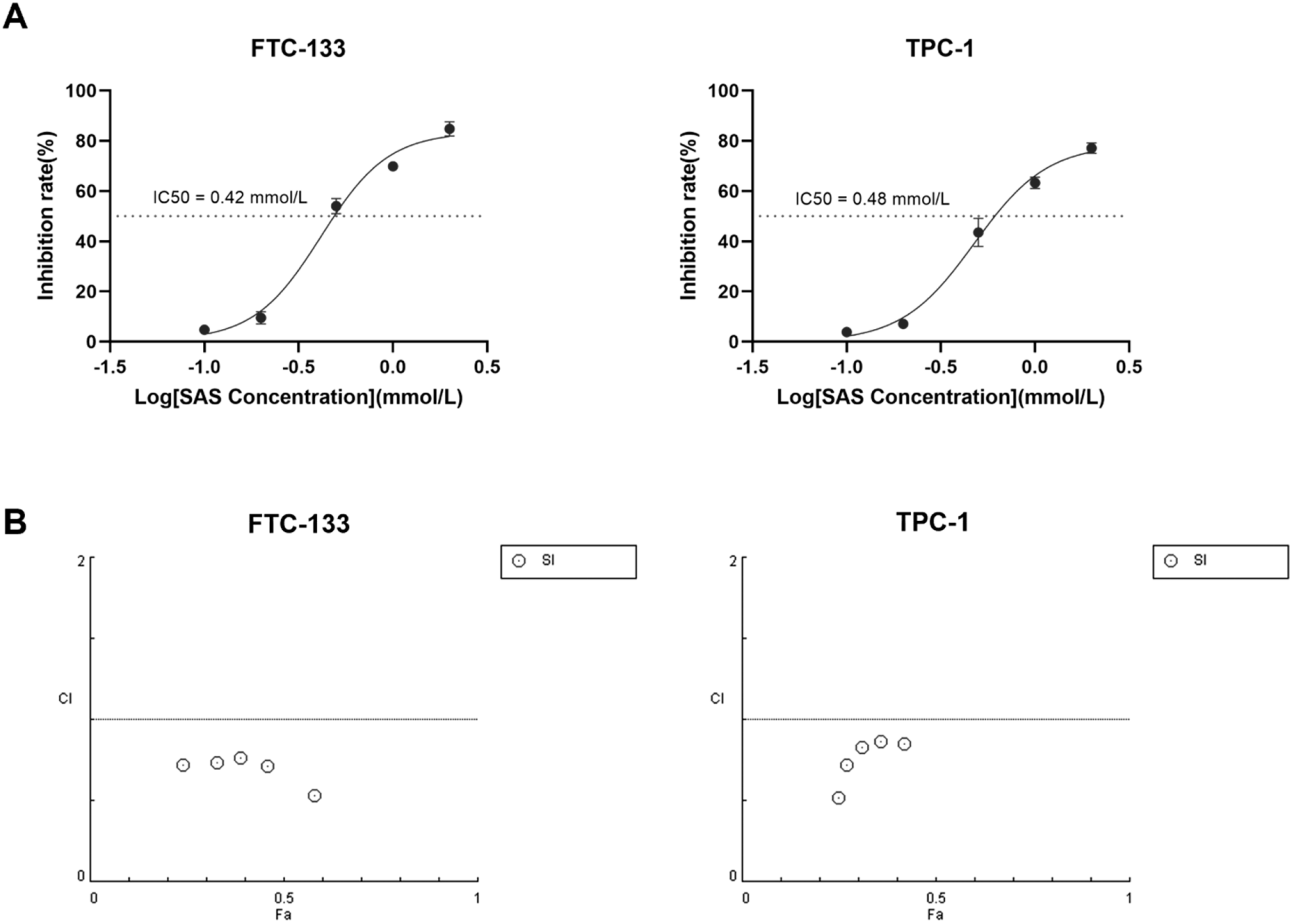
SAS synergizes with ^131^I to promote DTC cells death **(A)** The IC50 of SAS on FTC-133 and TPC-1 cells for 24 h. **(B)** Combination Index of sulfasalazine and ^131^I on FTC-133 and TPC-1 cells (concentration of SAS when treating alone on the cell: 0.1 mM, 0.2 mM, 0.5 mM, 1 mM, 2 mM, concentration of SAS in combination on cells: 0.1 mM, dose of ^131^I when treating alone and in combination on cells: 5 μCi, 10 μCi, 15 μCi, 20 μCi, 25 μCi, drug treatment time: 24 h). Images are representative of at least three independent experiments.

Given the observed role of *SLC7A11* suppression in ^131^I-induced ferroptosis, we investigated whether pharmacological *SLC7A11* inhibition could enhance ^131^I efficacy in DTC cells. The system xc^-^ inhibitor SAS, a class 1 ferroptosis inducer targeting *SLC7A11*, was selected for combination therapy. Dose-response analysis determined IC50 values of 0.42 mM for FTC-133 and 0.48 mM for TPC-1 cells (**Figure 4A**), prompting selection of sub-IC50 SAS concentrations (0.1 mM) for synergy testing. DTC cells were treated with escalating ^131^I doses (5, 10, 15, 20, 25 μCi) alone, 0.1 mM SAS monotherapy, or their combination. Combination Index (CI) was calculated using the Chou - Talalay method (28), where CI > 1 indicates antagonism; = 1 additive effect; < 1 synergy. Quantitative analysis demonstrated the CI values for the combination of SAS and ^131^I in DTC cells were less than 1, confirming synergistic effects between SAS and ^131^I in DTC cells (**Figure 4B**). Additionally, our findings indicated that the combined use of SAS and ^131^I also inhibits the activity of normal thyroid cells. However, compared to normal thyroid cells, the combined treatment exhibits a significantly more pronounced inhibitory effect on thyroid cancer cells, suggesting a potential therapeutic advantage in thyroid cancer cells while minimizing the toxic effect on healthy tissue (**Supplementary Figure S1**).

### Combination SAS and ^131^I enhance ferroptosis in DTC cells

**Figure 5:**
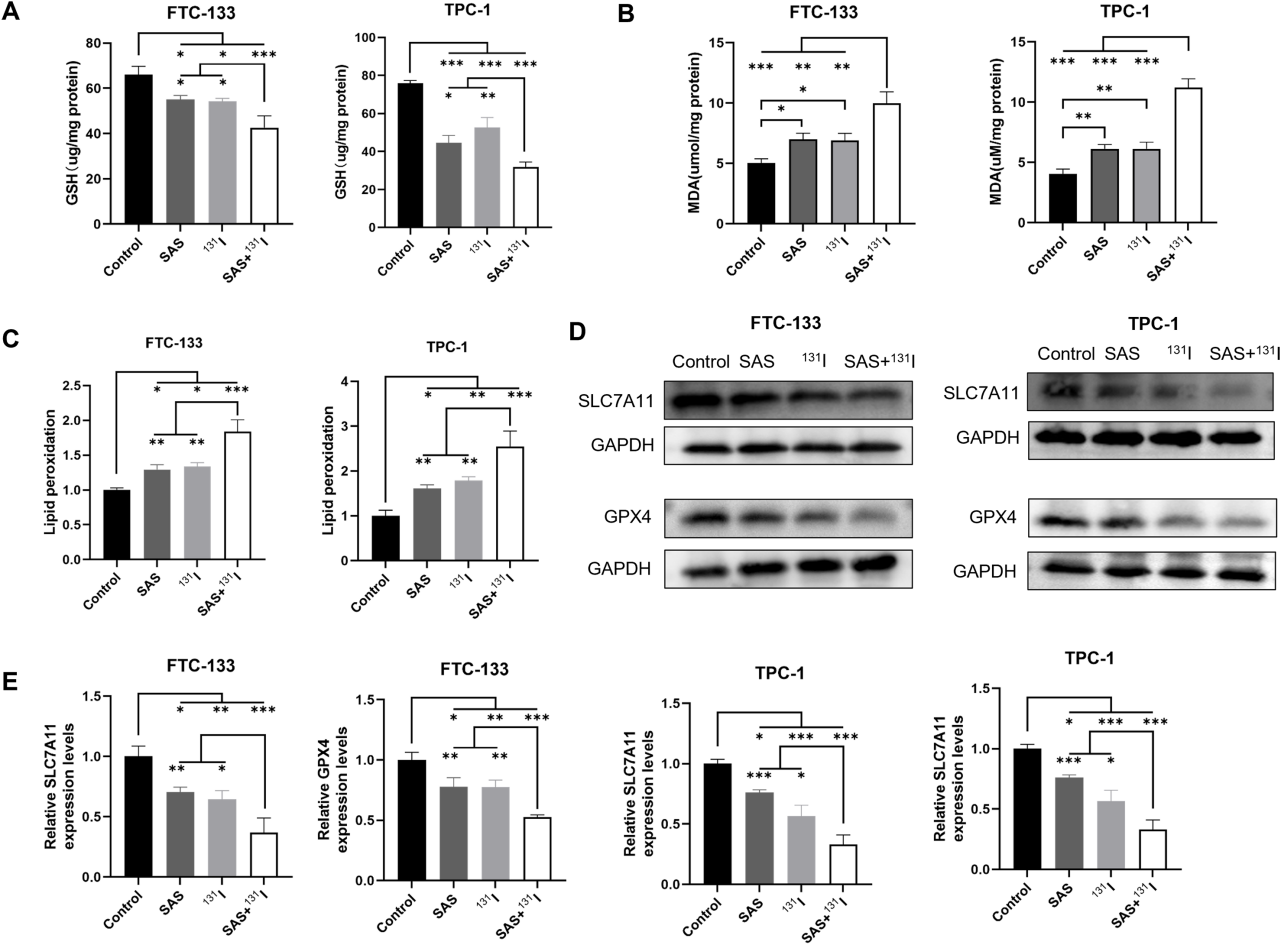
Combination SAS and ^131^I enhance lipid peroxidation and ferroptosis in DTC cells. **(A)** The GSH levels of FTC-133 and TPC-1 cells after 0.1 mmol/L SAS and 50 μCi ^131^I treatment for 24 h. **(B)** The MDA levels of FTC-133 and TPC-1 cells after 0.1 mM SAS and 50 μCi ^131^I treatment for 24 h. **(C)** The lipid peroxidation levels of FTC-133 and TPC-1 cells after 0.1 mM SAS and 50 μCi ^131^I treatment for 24 h. **(D)** The *GPX4* and *SLC7A11* protein expression levels of FTC-133 and TPC-1 cells after 0.1 mM SAS and 50 μCi ^13^1I treatment for 24 h. **(E)** The quantitative graph of relative protein expression of *SLC7A11* and *GPX4* using Image J. Images are representative of at least three independent experiments.

To mechanistically delineate the synergistic effects of SAS and ^131^I combination therapy in DTC cells, we quantified canonical ferroptosis biomarkers (MDA, GSH and lipid peroxidation) using standardized protocols described above. Intriguingly, the combination group exhibited significantly GSH depletion in FTC-133 and TPC-1 cells compared with the other groups (**Figure 5A**). And the cotreatment with SAS and ^131^I resulted in a marked elevation in MDA levels compared to the other treatment groups (**Figure 5B**). Furthermore, the combination of SAS and ^131^I resulted in a significant induction of lipid peroxidation, which was significantly higher than that in the other groups (**Figure 5C**). Moreover, we examined the protein expression levels of GPX4 and SLC7A11 in FTC-133 and TPC-1 cells using Western Blotting. Our results showed that GPX4 and SLC7A11 expression levels were significantly lower in the group treated with the SAS and ^131^I combination than in those treated with SAS alone, ^131^I alone, or in the control group. Additionally, GPX4 and SLC7A11 expression levels were significantly downregulated in groups treated with ^131^I or SAS alone compared to the control group. (**Figures 5D, E**). The above results indicate that cotreatment with SAS and ^131^I robustly induces cell death through enhanced lipid peroxidation and activation of ferroptosis.

### *SLC7A11* knockdown enhances ferroptosis induced by ^131^I and SAS in DTC cells

**Figure 6:**
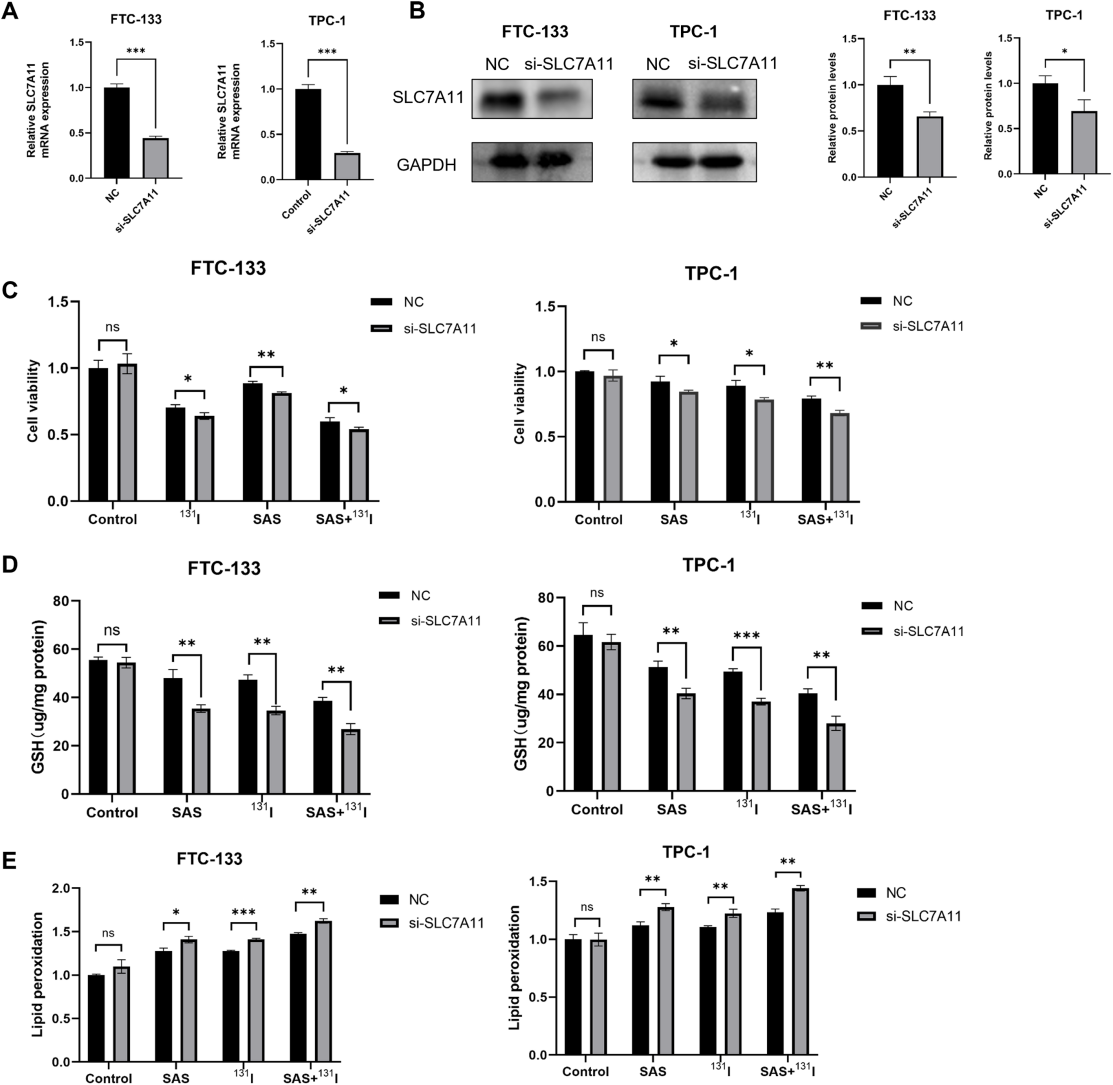
*SLC7A11* knockdown enhances effects of SAS and ^131^I on DTC cells. **(A)** Relative expression levels of *SLC7A11* mRNA in FTC-133 and TPC-1 cells with or without knockdown of *SLC7A11*. **(B)** Relative *SLC7A11* protein expression levels in FTC-133 and TPC-1 cells with or without knockdown of *SLC7A11*. **(C)** Cell viability of DTC FTC-133 and TPC-1 cells treated with 0.1 mmol/L sulfasalazine and 10 μCi ^131^I for 24 h with or without knockdown of *SLC7A11*. **(D)** GSH levels of FTC-133 and TPC-1 cells treated with 0.1 mmol/L sulfasalazine and 50 μCi ^131^I for 24 h with or without knockdown of *SLC7A11*. **(E)** Lipid peroxidation levels of FTC-133 and TPC-1 cells treated with 0.1 mmol/L sulfasalazine and 50 μCi ^131^I for 24 h with or without knockdown of *SLC7A11*. Images are representative of at least three independent experiments.

Given that the mechanism of action of SAS involves the inhibition of *SLC7A11* activity, and our study has also demonstrated that ^131^I can downregulate the expression of *SLC7A11*, we posited that their synergistic effects arose from dual targeting of this cystine/glutamate antiporter. To validate this hypothesis, we established *SLC7A11*-knockdown DTC cell models, validated by RT-qPCR and Western blotting (**Figures 6A, B**). To further elucidate the role of *SLC7A11* in mediating the effects of ^131^I and SAS on DTC cells, we assessed cell viability, intracellular GSH levels, and lipid peroxidation in *SLC7A11*-knockdown DTC cells. Our results demonstrated that *SLC7A11* knockdown significantly reduced cell viability, irrespective of whether ^131^I, SAS, or their combination was applied (**Figure 6C**). Additionally, *SLC7A11* knockdown led to a marked decrease in intracellular GSH levels across all treatment groups, except for the control group (**Figure 6D**). Moreover, lipid peroxidation levels were significantly elevated in cells treated with ^131^I alone, SAS alone, or the combination therapy following *SLC7A11* knockdown (**Figure 6E**). Collectively, these findings highlight the pivotal role of *SLC7A11* in the cytotoxic effects of ^131^I and SAS on thyroid cancer cells.

### SAS synergizes with ^131^I in DTC xenograft nude mice model

**Figure 7:**
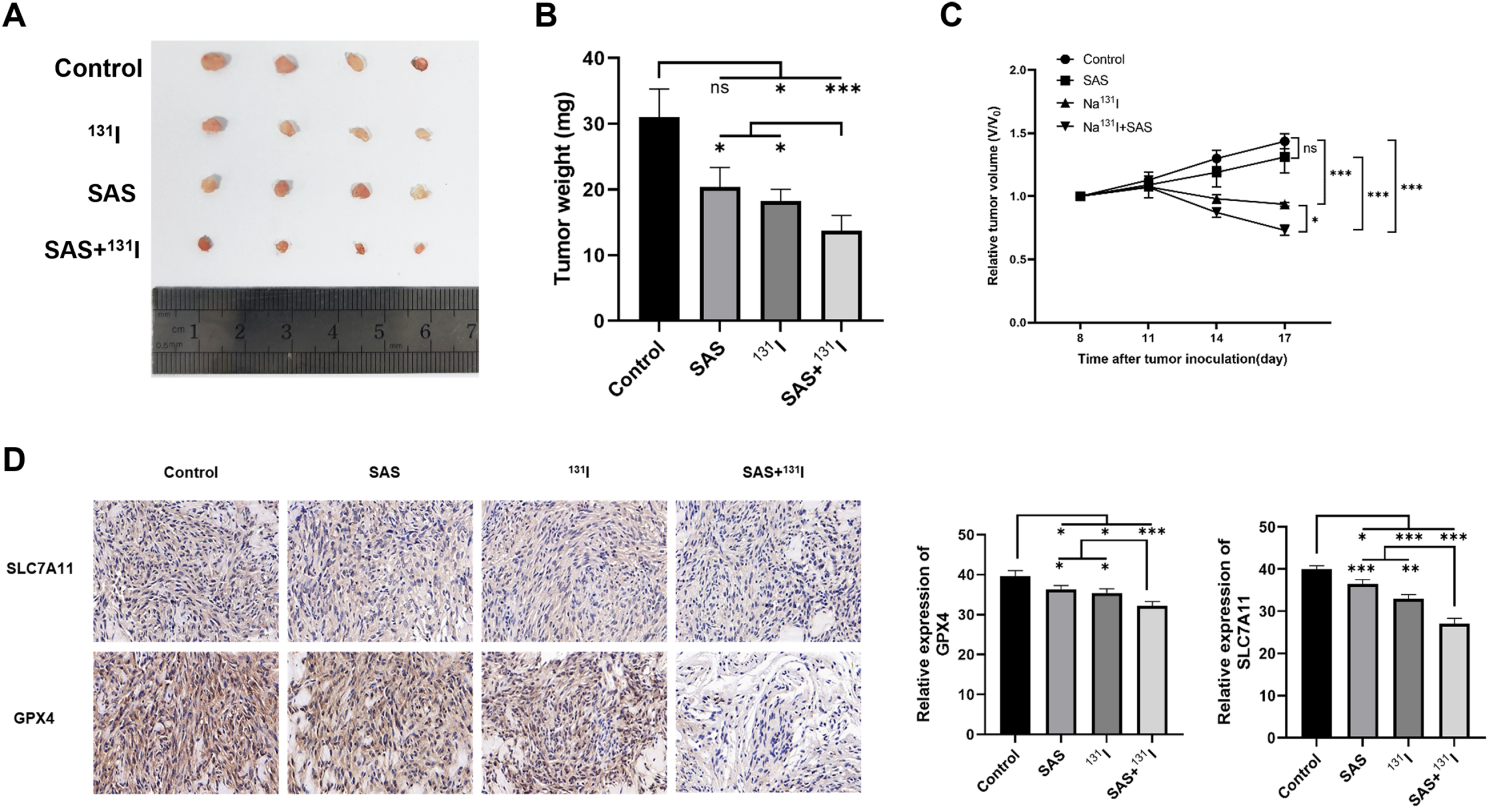
SAS synergizes with ^131^I in DTC xenograft nude mice model. **(A)** Image of tumors isolated from different treatment groups. **(B)** Tumor weights in different treatment groups. **(C)** Curves of relative tumor volume changes in different treatment groups during the treatment period. **(D)** Immunohistochemical analysis of *SLC7A11* and *GPX4*.

To validate the therapeutic synergy of SAS and ^131^I *in vivo*, TPC-1 xenograft-bearing nude mice were treated with SAS in combination with ^131^I. Post-treatment, significant reductions in tumor volume and weight were observed in the combination-treated group compared to the control group (**Figures 7A-C**). Additionally, treatment with ^131^I alone also resulted in notable decreases in tumor volume and weight compared to the control group. Immunohistochemical analyses revealed that the combination of ^131^I and SAS significantly downregulated the expression of GPX4 and SLC7A11 compared to all other group *in vivo* (**Figure 7D**). Moreover, GPX4 and SLC7A11 expression levels were also reduced in mice treated with ^131^I alone or SAS alone compared to the control group. Collectively, these findings suggest that SAS synergized with ^131^I to effectively treat DTC *in vivo*, an effect that is associated with enhanced lipid peroxidation and ferroptosis.

## Discussion

Ferroptosis, an iron-dependent form of regulated cell death driven by lipid peroxidation (29), functions as a tumor-suppressive mechanism that is frequently silenced in malignancies (11). Emerging evidence links multiple frontline oncologic therapies—including radiotherapy, endocrine therapy, immune checkpoint blockade, and platinum-based chemotherapy—to ferroptosis potentiation (15, 30–32). The synergistic application of ferroptosis inducer in combination with other therapeutic approaches represents a promising strategy for cancer therapy (33, 34). In this study, we demonstrated that ^131^I induces lipid peroxidation and ferroptosis in DTC cells and synergizes with SAS in treating DTC cells. Fer-1 alleviated the inhibitory effects of ^131^I on the viability and clonogenic potential of DTC cells. Through a comprehensive analysis encompassing biochemical, morphological, and genetic perspectives, we have identified that ^131^I induces ferroptosis in DTC cells. Specifically, ^131^I treatment results in elevated levels of MDA and ROS, concurrent with a reduction in GSH content within DTC cells. Additionally, ^131^I induces characteristic morphological changes associated with ferroptosis, including a reduction in mitochondrial volume and contraction of mitochondrial cristae. Furthermore, the expression levels of *SLC7A11* and *GPX4* are downregulated in DTC cells after ^131^I treatment.

As a radionuclide,^131^I emits β radiation that induces cell death by damaging DNA and eliciting cytotoxicity. The presence of the NIS on the basal membrane of certain tumor cells, such as those in DTC, cholangiocarcinoma and hepatocellular carcinoma, facilitates iodine uptake and forms the basis for radioiodine therapy (35). However, ^131^I can be used for the radiolabeling of molecules, peptides, antibodies and nanomaterials that selectively binding to cancer cells, thereby targeting cancer cells that do not express NIS. For instance, a ^131^I-labeled programmed cell death ligand 1 (PD-L1) antibody can target PD-L1 expressed on the cancer cell membrane, inducing ferroptosis in cancer cells (36). Two studies on the synthesis of radiopharmaceuticals using ^131^I-conjugated PD-L1 antibodies have demonstrated that these radiotherapeutic agents can induce ferroptosis in tumor cells (36, 37). Moreover, Shen et al. employed 3 nm ultrasmall single-crystal iron nanoparticles as a ferroptosis inducer to synergize with ^131^I-PD-L1 antibodies in treating murine breast cancer cells and embryonic fibroblast cells. These findings support our investigation that ^131^I can induce lipid peroxidation and ferroptosis in cancer cells. However, given the inability of these cells to uptake ^131^I, experiments on the therapeutic application of ^131^I as a solitary agent were not conducted in their research. By contrast, the DTC cells used in the present study are capable of iodine uptake, allowing us to directly observe the role of ferroptosis in tumor cells treated with ^131^I.

Extensive scholarly research has demonstrated that the induction of ferroptosis enhances the efficacy of radiotherapy in cancer treatment, and can even overcomes the radioresistance of some cancer cells (34, 38–40). SAS has been shown to induce ferroptosis by inhibiting the SLC7A11 transporter, a transmembrane protein responsible for cysteine uptake into cells (41). This inhibition leads to a depletion of GSH, a coenzyme essential for the function of GPX4. Previous studies have identified that SAS exhibits significant radiosensitizing effect both *in vivo* and *in vitro* (14, 20, 42). However, no research has yet explored whether SAS can synergistically treat DTC by enhancing the ferroptosis-inducing effects of ^131^I. In the current study, we treated DTC cells with a combination of ^131^I with SAS, and observed that the CI was below 1, indicative of a synergistic interaction. Additionally, cotreatment resulted in the depletion of GSH and the elevation of ROS and MDA levels in DTC cells, along with a marked decrease in the expression of *SLC7A11* and *GPX4*. Our study confirmed that *SLC7A11* is downregulated in DTC cells treated with ^131^I. Furthermore, SAS induces ferroptosis by inhibiting the activity of *SLC7A11*. Therefore, we propose that *SLC7A11* plays a crucial role in the synergistic effects of ^131^I and SAS on DTC cells. Accordingly, in this study, we knocked down the expression of *SLC7A11* in TPC-1 and FTC-133 cells and subsequently examined the changes in ferroptosis-related markers following exposure to ^131^I. The results revealed that, compared with cells without knockdown, cells with *SLC7A11* knockdown exhibited more pronounced ferroptosis-related responses, regardless of whether they were treated with ^131^I alone, SAS alone, or a combination of both agents. Furthermore, this study also demonstrated that SAS and ^131^I can exert synergistic antitumor effects through inducing ferroptosis *in vivo*. Overall, we suggest that SAS and ^131^I can synergistically induce lipid peroxidation and ferroptosis by depleting GSH through the inhibition of *SLC7A11*.

While our study demonstrates the potential of combining ^131^I and SAS to induce ferroptosis in DTC, we acknowledge that the effects of this combination therapy on normal thyroid cells and GPX4 or SLC7A11 overexpression experiments on DTC cells were not explored. Although ^131^I is a standard treatment for DTC with a well-established safety profile, and SAS selectively targets ferroptosis in cancer cells, the toxicity of this combination therapy on normal thyroid tissue remains an important consideration. Normal thyroid cells typically express lower levels of GPX4 and SLC7A11 compared to DTC cells, which may reduce their susceptibility to ferroptosis induction (22–25). However, further comparison and *in vivo* studies are required to assess the potential toxicity of ^131^I and SAS on normal thyroid cells and to investigate effects of GPX4 or SLC7A11 overexpression on DTC cells, ensuring that this combination therapy can be applied safely within a therapeutic window. Additionally, given the crucial role of the NIS in iodine uptake by thyroid cancer cells, future research should focus on whether and how SAS affects NIS expression. This is vital for understanding the synergistic effects of combining ^131^I and SAS in DTC cells.

## Conclusions

Our study highlights that the combination of ^131^I and SAS can synergistically induce lipid peroxidation and ferroptosis in DTC cells, leading to enhanced therapeutic efficacy. This suggests that targeting ferroptosis pathways, in conjunction with traditional therapies like ^131^I, may provide a promising approach to improve the treatment of DTC.

## Data Availability

The raw data supporting the conclusions of this article will be made available by the authors, without undue reservation.

## References

[1] Bray F Laversanne M Sung H Ferlay J Siegel RL Soerjomataram I. Global cancer statistics 2022: GLOBOCAN estimates of incidence and mortality worldwide for 36 cancers in 185 countries. CA Cancer J Clin. (2024) 74:229–63. doi: 10.3322/caac.21834 38572751

[2] Sung H Ferlay J Siegel RL Laversanne M Soerjomataram I Jemal A. Global cancer statistics 2020: GLOBOCAN estimates of incidence and mortality worldwide for 36 cancers in 185 countries. CA Cancer J Clin. (2021) 71:209–49. doi: 10.3322/caac.21660 33538338

[3] Boucai L Zafereo M Cabanillas ME. Thyroid cancer: A review. JAMA. (2024) 331:425–35. doi: 10.1001/jama.2023.26348 38319329

[4] Ao Z-X Chen Y-C Lu J-M Shen J Peng L-P Lin X. Identification of potential functional genes in papillary thyroid cancer by co-expression network analysis. Oncol Lett. (2018) 16:4871–8. doi: 10.3892/ol.2018.9306 PMC6144229 30250553

[5] Br H Ek A Kc B Gm D Sj M Ye N. 2015 American thyroid association management guidelines for adult patients with thyroid nodules and differentiated thyroid cancer: the american thyroid association guidelines task force on thyroid nodules and differentiated thyroid cancer. Thyroid Off J Am Thyroid Assoc. (2016) 26. doi: 10.1089/thy.2015.0020 PMC4739132 26462967

[6] Dixon SJ Lemberg KM Lamprecht MR Skouta R Zaitsev EM Gleason CE. Ferroptosis: an iron-dependent form of non-apoptotic cell death. Cell. (2012) 149:1060–72. doi: 10.1016/j.cell.2012.03.042 PMC3367386 22632970

[7] Li Y Feng D Wang Z Zhao Y Sun R Tian D. Ischemia-induced ACSL4 activation contributes to ferroptosis-mediated tissue injury in intestinal ischemia/reperfusion. Cell Death Differ. (2019) 26:2284–99. doi: 10.1038/s41418-019-0299-4 PMC6889315 30737476

[8] Cai W Liu L Shi X Liu Y Wang J Fang X. Alox15/15-hpETE aggravates myocardial ischemia-reperfusion injury by promoting cardiomyocyte ferroptosis. Circulation. (2023) 147:1444–60. doi: 10.1161/CIRCULATIONAHA.122.060257 36987924

[9] Luoqian J Yang W Ding X Tuo Q-Z Xiang Z Zheng Z. Ferroptosis promotes T-cell activation-induced neurodegeneration in multiple sclerosis. Cell Mol Immunol. (2022) 19:913–24. doi: 10.1038/s41423-022-00883-0 PMC9338013 35676325

[10] Chu J Li J Sun L Wei J. The role of cellular defense systems of ferroptosis in Parkinson’s disease and Alzheimer’s disease. Int J Mol Sci. (2023) 24. doi: 10.3390/ijms241814108 PMC10531775 37762411

[11] Jiang L Kon N Li T Wang S-J Su T Hibshoosh H. Ferroptosis as a p53-mediated activity during tumour suppression. Nature. (2015) 520:57–62. doi: 10.1038/nature14344 25799988 PMC4455927

[12] Li D Wang Y Dong C Chen T Dong A Ren J. CST1 inhibits ferroptosis and promotes gastric cancer metastasis by regulating GPX4 protein stability via OTUB1. Oncogene. (2023) 42. doi: 10.1038/s41388-022-02537-x PMC9816059 36369321

[13] Zhou Q Liu T Qian W Ji J Cai Q Jin Y. HNF4A-BAP31-VDAC1 axis synchronously regulates cell proliferation and ferroptosis in gastric cancer. Cell Death Dis. (2023) 14:356. doi: 10.1038/s41419-023-05868-z 37296105 PMC10256786

[14] Lei G Zhang Y Koppula P Liu X Zhang J Lin SH. The role of ferroptosis in ionizing radiation-induced cell death and tumor suppression. Cell Res. (2020) 30:146–62. doi: 10.1038/s41422-019-0263-3 PMC7015061 31949285

[15] Lang X Green MD Wang W Yu J Choi JE Jiang L. Radiotherapy and immunotherapy promote tumoral lipid oxidation and ferroptosis via synergistic repression of SLC7A11. Cancer Discov. (2019) 9:1673–85. doi: 10.1158/2159-8290.CD-19-0338 PMC6891128 31554642

[16] Bersuker K Hendricks JM Li Z Magtanong L Ford B Tang PH. The CoQ oxidoreductase FSP1 acts parallel to GPX4 to inhibit ferroptosis. Nature. (2019) 575:688–92. doi: 10.1038/s41586-019-1705-2 PMC6883167 31634900

[17] Koppula P Zhuang L Gan B. Cystine transporter SLC7A11/xCT in cancer: ferroptosis, nutrient dependency, and cancer therapy. Protein Cell. (2021) 12:599–620. doi: 10.1007/s13238-020-00789-5 33000412 PMC8310547

[18] Yang WS SriRamaratnam R Welsch ME Shimada K Skouta R Viswanathan VS. Regulation of ferroptotic cancer cell death by GPX4. Cell. (2014) 156:317–31. doi: 10.1016/j.cell.2013.12.010 PMC4076414 24439385

[19] Gout PW Buckley AR Simms CR Bruchovsky N. Sulfasalazine, a potent suppressor of lymphoma growth by inhibition of the x(c)- cystine transporter: a new action for an old drug. Leukemia. (2001) 15:1633–40. doi: 10.1038/sj.leu.2402238 11587223

[20] Nagane M Kanai E Shibata Y Shimizu T Yoshioka C Maruo T. Sulfasalazine, an inhibitor of the cystine-glutamate antiporter, reduces DNA damage repair and enhances radiosensitivity in murine B16F10 melanoma. PloS One. (2018) 13:e0195151. doi: 10.1371/journal.pone.0195151 29649284 PMC5896924

[21] Rodman SN Spence JM Ronnfeldt TJ Zhu Y Solst SR O’Neill RA. Enhancement of radiation response in breast cancer stem cells by inhibition of thioredoxin and glutathione dependent metabolism. Radiat Res. (2016) 186:385–95. doi: 10.1667/RR14463.1 PMC5077643 27643875

[22] Shen L Qian C Cao H Wang Z Luo T Liang C. Upregulation of the solute carrier family 7 genes is indicative of poor prognosis in papillary thyroid carcinoma. World J Surg Oncol. (2018) 16:235. doi: 10.1186/s12957-018-1535-y 30558624 PMC6297957

[23] Chen H Peng F Xu J Wang G Zhao Y. Increased expression of GPX4 promotes the tumorigenesis of thyroid cancer by inhibiting ferroptosis and predicts poor clinical outcomes. Aging. (2023) 15:230–45. doi: 10.18632/aging.204473 PMC9876627 36626251

[24] Sekhar KR Hanna DN Cyr S Baechle JJ Kuravi S Balusu R. Glutathione peroxidase 4 inhibition induces ferroptosis and mTOR pathway suppression in thyroid cancer. Sci Rep. (2022) 12:19396. doi: 10.1038/s41598-022-23906-2 36371529 PMC9653479

[25] Ji F-H Fu X-H Li G-Q He Q Qiu X-G. FTO prevents thyroid cancer progression by SLC7A11 m6A methylation in a ferroptosis-dependent manner. Front Endocrinol. (2022) 13:857765. doi: 10.3389/fendo.2022.857765 PMC9205202 35721711

[26] Yang WS Stockwell BR. Ferroptosis: death by lipid peroxidation. Trends Cell Biol. (2016) 26:165–76. doi: 10.1016/j.tcb.2015.10.014 PMC4764384 26653790

[27] Stockwell BR Friedmann Angeli JP Bayir H Bush AI Conrad M Dixon SJ. Ferroptosis: A regulated cell death nexus linking metabolism, redox biology, and disease. Cell. (2017) 171:273–85. doi: 10.1016/j.cell.2017.09.021 PMC5685180 28985560

[28] Tc C. Drug combination studies and their synergy quantification using the Chou-Talalay method. Cancer Res. (2010) 70. doi: 10.1158/0008-5472.CAN-09-1947 20068163

[29] Stockwell BR. Ferroptosis turns 10: Emerging mechanisms, physiological functions, and therapeutic applications. Cell. (2022) 185:2401–21. doi: 10.1016/j.cell.2022.06.003 PMC9273022 35803244

[30] Wang W Green M Choi JE Gijón M Kennedy PD Johnson JK. CD8+ T cells regulate tumour ferroptosis during cancer immunotherapy. Nature. (2019) 569:270–4. doi: 10.1038/s41586-019-1170-y PMC6533917 31043744

[31] Xu Z Wang X Sun W Xu F Kou H Hu W. RelB-activated GPX4 inhibits ferroptosis and confers tamoxifen resistance in breast cancer. Redox Biol. (2023) 68:102952. doi: 10.1016/j.redox.2023.102952 37944384 PMC10641764

[32] Guo J Xu B Han Q Zhou H Xia Y Gong C. Ferroptosis: A novel anti-tumor action for cisplatin. Cancer Res Treat. (2018) 50:445–60. doi: 10.4143/crt.2016.572 PMC5912137 28494534

[33] Wang L Wang C Li X Tao Z Zhu W Su Y. Melatonin and erastin emerge synergistic anti-tumor effects on oral squamous cell carcinoma by inducing apoptosis, ferroptosis, and inhibiting autophagy through promoting ROS. Cell Mol Biol Lett. (2023) 28:36. doi: 10.1186/s11658-023-00449-6 37131152 PMC10155313

[34] Ye LF Chaudhary KR Zandkarimi F Harken AD Kinslow CJ Upadhyayula PS. Radiation-induced lipid peroxidation triggers ferroptosis and synergizes with ferroptosis inducers. ACS Chem Biol. (2020) 15:469–84. doi: 10.1021/acschembio.9b00939 PMC7180072 31899616

[35] Shen H Zhu R Liu Y Hong Y Ge J Xuan J. Radioiodine-refractory differentiated thyroid cancer: molecular mechanisms and therapeutic strategies for radioiodine resistance. Drug Resist Update Rev Comment Antimicrob Anticancer Chemother. (2024) 72:101013. doi: 10.1016/j.drup.2023.101013 38041877

[36] Shao C Yan X Pang S Nian D Ren L Li H. Bifunctional molecular probe targeting tumor PD-L1 enhances anti-tumor efficacy by promoting ferroptosis in lung cancer mouse model. Int Immunopharmacol. (2024) 130:111781. doi: 10.1016/j.intimp.2024.111781 38442580

[37] Shen J Feng K Yu J Zhao Y Chen R Xiong H. Responsive and traceless assembly of iron nanoparticles and 131I labeled radiopharmaceuticals for ferroptosis enhanced radio-immunotherapy. Biomaterials. (2025) 313:122795. doi: 10.1016/j.biomaterials.2024.122795 39232333

[38] Lei G Zhang Y Hong T Zhang X Liu X Mao C. Ferroptosis as a mechanism to mediate p53 function in tumor radiosensitivity. Oncogene. (2021) 40:3533–47. doi: 10.1038/s41388-021-01790-w PMC8141034 33927351

[39] Zeng L Ding S Cao Y Li C Zhao B Ma Z. A MOF-based potent ferroptosis inducer for enhanced radiotherapy of triple negative breast cancer. ACS Nano. (2023) 17:13195–210. doi: 10.1021/acsnano.3c00048 37256771

[40] Liu S Zhang H-L Li J Ye Z-P Du T Li L-C. Tubastatin A potently inhibits GPX4 activity to potentiate cancer radiotherapy through boosting ferroptosis. Redox Biol. (2023) 62:102677. doi: 10.1016/j.redox.2023.102677 36989572 PMC10074938

[41] Ma M-Z Chen G Wang P Lu W-H Zhu C-F Song M. Xc- inhibitor sulfasalazine sensitizes colorectal cancer to cisplatin by a GSH-dependent mechanism. Cancer Lett. (2015) 368:88–96. doi: 10.1016/j.canlet.2015.07.031 26254540

[42] Kerkhove L Geirnaert F Rifi AL Law KL Gutiérrez A Oudaert I. Repurposing sulfasalazine as a radiosensitizer in hypoxic human colorectal cancer. Cancers. (2023) 15:2363. doi: 10.3390/cancers15082363 37190291 PMC10137052

