# Supplementary figures and images for: Iodine-131 induces ferroptosis and synergizes with sulfasalazine in differentiated thyroid cancer cells via suppressing SLC7A11
Source: Front Oncol. 2025 May 19;15:1580828. doi: 10.3389/fonc.2025.1580828 (PMC12127297; doi:10.3389/fonc.2025.1580828)

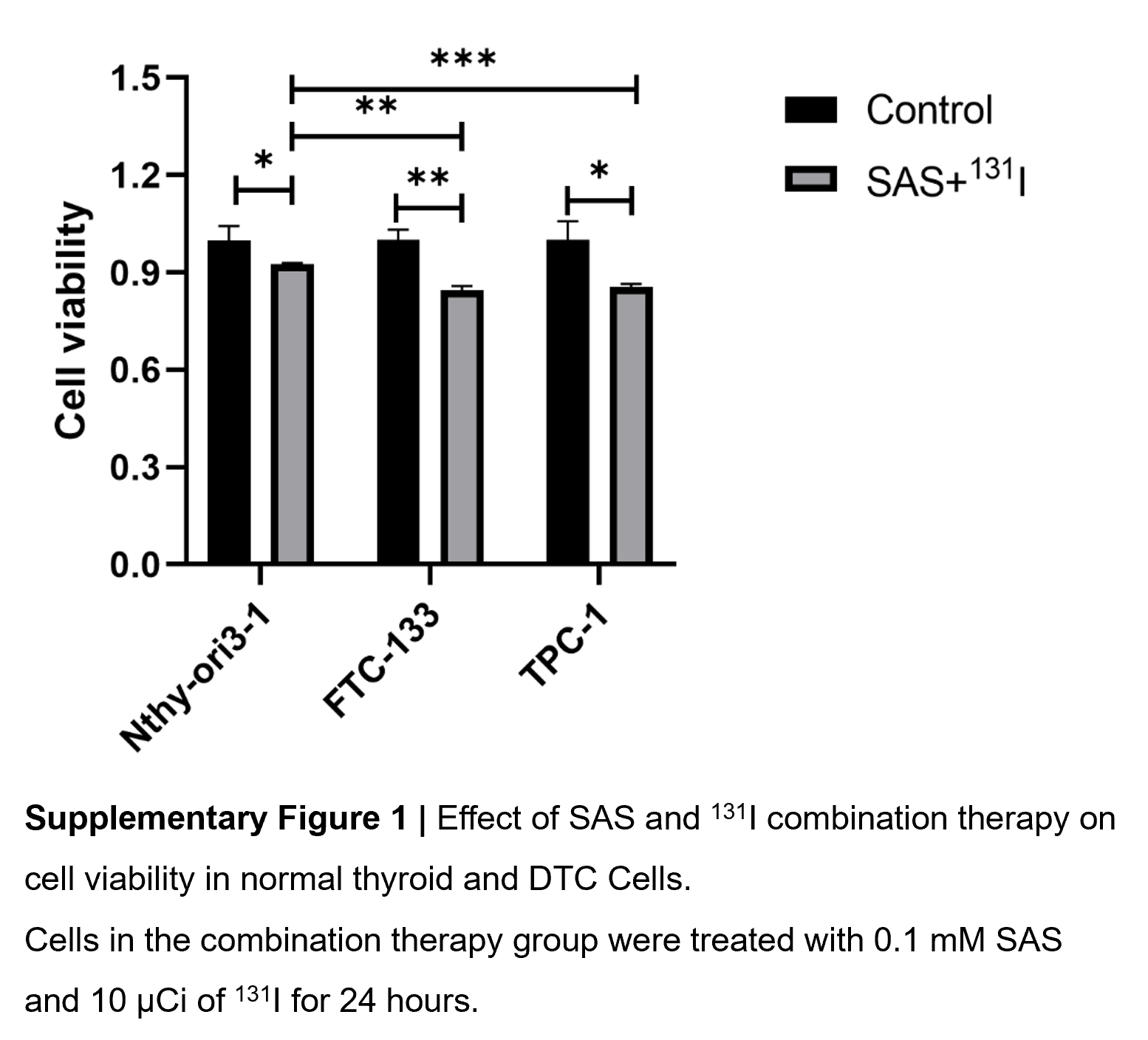

Supplement: Supplementary file 1 [file Image1.tif]
